# Mitochondrial Transplantation Increases Bioenergetics and Neurite Outgrowth in Healthy and P301Ltau-Expressing SH-SY5Y Cells

**DOI:** 10.1007/s12035-025-05604-y

**Published:** 2025-12-10

**Authors:** Aline Broeglin, Aurélien Riou, Andreas Papassotiropoulos, Anne Eckert, Amandine Grimm

**Affiliations:** 1 Research Cluster, Molecular & Cognitive Neuroscience, Department of Biomedicine, University of Basel, Basel, Switzerland; 2 Neurobiology Laboratory for Brain Aging and Mental Health, University Psychiatric Clinics (UPK), Basel, Switzerland

**Keywords:** Tauopathies, P301Ltau mutation, Mitochondria, Transplantation, Bioenergetic, Neurites

## Abstract

Tauopathies are neurodegenerative diseases characterized by the abnormal accumulation of tau protein in neurons, leading to cognitive impairment. A common feature of these disorders is mitochondrial dysfunction, which leads to bioenergetic deficits and contributes to neuronal cell death. As neurons have high energy demands, impaired mitochondrial function directly affects their viability and function. Thus, mitochondria represent an attractive target for neuroprotective strategies in tauopathies. Mitochondrial transplantation (MT) is an emerging therapeutic approach to restoring cellular bioenergetics. Although MT has shown promise in various models of brain diseases, its efficacy has not been evaluated in the context of tau-induced mitochondrial dysfunction. This study examines the impact of MT on healthy cells and in a cellular model of tauopathy. Mitochondria were freshly isolated from astrocytic cells and transplanted into healthy SH-SY5Y neuroblastoma cells and SH-SY5Y cells overexpressing the P301L tau mutation, for 24 and 48 h. Our results demonstrate that MT enhances cell viability, ATP production, mitochondrial membrane potential, and respiration in both healthy and tau-mutant SH-SY5Y cells. In addition, MT reduced mitochondrial superoxide anion levels and promoted neurite outgrowth in both cell lines. Key bioenergetic outcomes were recapitulated in neurons derived from induced pluripotent stem cells (iPSCs) carrying the P301L tau mutation. These findings suggest that MT might be a promising therapeutic strategy to counteract mitochondrial deficits in tauopathies. Importantly, this approach positions mitochondria not as a target but as the therapeutic agent itself. Further studies are warranted to advance MT toward in vivo applications in tau-related neurodegenerative disorders.

**Supplementary Information:**

The online version contains supplementary material available at 10.1007/s12035-025-05604-y.

## Introduction

Neurodegenerative diseases are primarily age-related and represent a significant public health concern. These conditions are marked by the progressive degeneration and loss of neuronal cells in both the central and peripheral nervous systems [1, 2]. Tauopathies are part of this group of neurodegenerative diseases. Notable examples include Alzheimer's disease (AD), frontotemporal dementia (FTD), progressive supranuclear palsy, and amyotrophic lateral sclerosis. The tau protein, encoded by the MAPT gene (Microtubule-Associated Protein Tau) on chromosome 17, plays a crucial role in stabilizing, assembling, and functioning of microtubules [3]. Furthermore, this protein facilitates several essential functions in neuronal cells, including axonal transport, neurotransmission, and cell polarity [4]. However, in a pathological context, tau protein undergoes abnormal hyperphosphorylation, which leads to intracellular tau accumulation and the formation of neurofibrillary tangles in the brain. The mechanisms responsible for tau-induced neuronal dysfunction remain unclear.

Studies have demonstrated that tau protein exhibits abnormal interactions with mitochondria, disrupting their cellular functions and contributing to neuronal death and loss [4, 5]. Mitochondria, also known as the "powerhouses of the cell," are essential organelles contributing to vital cellular functions. They are not only required to ensure the production of primary cellular energy through the generation of adenosine triphosphate (ATP), but they are also involved in a wide range of functions, such as calcium buffering and metabolite synthesis [4]. Mitochondria are essential in neurons, which require approximately 15% of the total body's energy to support processes such as neurotransmitter release, action potential generation, and synaptic plasticity [6]. Given the pivotal role of mitochondria in these post-mitotic and highly differentiated cells, the impact of mitochondrial defects on brain functions is a salient consideration.

In tauopathies, the abnormal interaction between mitochondria and tau protein results in the impairment of mitochondrial morphology, such as mitochondrial swelling, and a reduction in their bioenergetic functions, including impaired ATP production and increased generation of reactive oxygen species (ROS) [7]. Furthermore, mitochondrial dynamics, particularly the balance between fusion and fission processes, are altered, resulting in an impairment of the mitochondrial recycling system, also known as mitophagy, which in turn leads to increased cellular stress [8, 9]. These mitochondrial impairments impact several cellular functions, including neuroplasticity, with a deficit observed in the neurite outgrowth process. Indeed, the abnormally hyperphosphorylated tau protein cannot stabilize the microtubules correctly, which is necessary for the neurite’s elongation. Additionally, it has been demonstrated that physiological tau protein can enhance specific pathways involved in initiating neurite elongation [10]. It is widely recognized that these mitochondrial dysfunctions often precede the cognitive deficits observed in neurodegenerative diseases [11]. Neurons, being post-mitotic cells, cannot divide and replenish their supply of mitochondria [6]. Therefore, neuronal mitochondria represent an interesting target for therapeutic intervention. Conventional therapeutic approaches typically involve enhancing mitochondrial bioenergetic function and mitigating mitochondrial oxidative damage. A new approach consists in using exogenous healthy mitochondria as a treatment. This approach is called mitochondrial transplantation.

This innovative technique involves isolating mitochondria from healthy donor cells and subsequently transferring them into recipient cells that exhibit mitochondrial dysfunction. It has been extensively documented in the context of cardiac diseases, where mitochondria also play a crucial role, as evidenced by the pioneering research conducted by the group of James McCully (Harvard Medical School, Boston, USA). They have demonstrated the efficacy of mitochondrial transplantation in enhancing cellular viability following ischemia–reperfusion injury in the heart [12]. An increasing number of research groups are exploring the potential of mitochondrial transplantation to treat disorders in various organs, including the kidneys, liver, and brain [13]. Mitochondrial transplantation has been tested in both in vitro and in vivo settings for several brain disorders, including traumatic brain injury, cognitive deficit, neurodegenerative diseases, and brain cancer (reviewed in [6]). Overall, encouraging results are observed after mitochondrial transplantation, notably including an increase in mitochondrial function, cellular viability, and an improvement in cognitive performance [14, 15]. However, no research has been conducted on the impact of mitochondrial transplantation in the context of tauopathies until today.

The specific aim of this study was to investigate the impact of mitochondrial transplantation on bioenergetic function and neurite outgrowth, particularly in the context of P301L tau expression. Indeed, we previously showed that P301L tau-overexpressing cells exhibit bioenergetic deficits and mitochondrial impairments in SH-SY5Y cells [16, 17]. The investigation of other hallmarks of tau pathology—such as tau hyperphosphorylation or aggregation—was beyond the scope of the present study and therefore was not addressed here.

We hypothesized that mitochondrial transplantation could enhance cellular viability, neurite outgrowth, and bioenergetic function in both healthy cells and cells overexpressing disease-associated P301Ltau protein. For this purpose, we used healthy SH-SY5Y cells and SH-SY5Y cells overexpressing the P301L tau mutation as a neuronal model. Given that intercellular mitochondrial transfer naturally occurs between glial cells and neurons—often yielding neuroprotective effects—an astrocytic cell line (A172) was used as a donor source of healthy mitochondria [18, 19].

In the first step, we investigated the effect of mitochondrial transplantation in healthy cells to ascertain the optimal parameters. Specifically, we evaluated the impact of mitochondrial transplantation on cell viability, ATP levels, mitochondrial membrane potential (MMP), cellular oxygen consumption rate (OCR), and reactive oxygen species (ROS) production. In the next step, we used the same approach and bioenergetic readouts with the "P301L-tau" SH-SY5Y cellular model of tauopathy. Key bioenergetic experiments were recapitulated in induced pluripotent stem cells (iPSCs) derived from a patient carrying the P301L tau mutation and differentiated into neurons. Finally, the study was completed with a neurite outgrowth investigation on the healthy and the P301L SH-SY5Y cells to assess the impact of mitochondria transplantation on neuronal morphology and differentiation.

## Materials and Methods

### Reagents/Chemicals

All the materials used for the study are listed in Table 1.

**Table 1:** List of materials and reagents used for the experiments

Reagent/ressource	Reference or source	Identifier or catalog number
Chemical, enzymes, materials		
Accutase	Innovative Cell Technologies, Inc	AT-104
ATPlite 1step Luminescence Assay System	Revvity Healthy Sciences Inc	6016943
Primary antibody anti-ß3 tubulin chicken	Abcam	ab41489
Secondary antibody anti-chicken coupled Alexa Fluor 488	Abcam	ab150173
Anti-beta III Tubulin (B3TUBB) antibody chicken	Abcam	ab41489
Anti-MAP2 antibody rabbit	Abcam	ab32454
Anti-EAAT1 antibody chicken	SYSY Synaptic System	250 116
Anti-S100B antibody guinea pig	SYSY Synaptic System	287 004
Anti-GFAP antibody rabbit	Abcam	ab68428
Goat Anti-Chicken IgY H&L (Alexa Fluor® 488) preadsorbed	Abcam	ab150173
Goat Anti-Rabbit IgG H&L (Alexa Fluor® 647) preadsorbed	Abcam	ab150083
Goat anti-rabbit IgG H&L (Alexa Fluor® 568)	Abcam	ab175471
Goat Anti-Guinea pig IgG H&L (Alexa Fluor® 647)	Abcam	ab150187
Invitrogen™Human Neural Stem Cell Immunocytochemistry Kit	Thermofisher	A24354
BDNF	PeproTech	**450–02**
Blasticidin	Invivogen	Ant-bl
BrdU kit	Merck	QIA58
BSA	Sigma-Aldrich	A9647
B-27	Gibco	17,504,001
Calcium chloride (CaCl_2_)	Sigma-Aldrich	449,709
Cell scraper	Sarstedt	83.3951
CellTracker Blue CMAC	Invitrogen	C2110
Culture dishes 10 cm^2^	Sarstedt	83.3902.300
Culture dises 20 cm^2^	Sarstedt	83.39.03
DAPI	Merck	10,236,276,001
DAPT	MedChem Express	HY-13027
DHE	Invitrogen	D11347
DMEM	Sigma-Aldrich	D6429
DMSO	Sigma-Aldrich	276,855
EDTA 0.5 M, pH 8.0 UltraPure™	Life Technologies	15,575,020
EGF	Sigma-Aldrich	E9644
EGTA	Thermoscientific	409,910,250
Fetal bovine serum	Corning	35–079-CV
FGF-2 (147)	LucernaChem	CYT-557
Filter 40 µm mesh size	PluriSelect	43–57,040-01
Filter 10 µm mesh size	PluriSelect	43–50,010-01
G418	Sigma-Aldrich	108,321–42-2
Geltrex™ LDEV-Free Reduced Growth Factor Basement Membrane Matrix	Thermofisher	A1413202
Gibco™ Essential 8™Medium	Thermofisher	A1517001
Gibco™ Advanced DMEM/F-12	Thermofisher	12,634,028
Gibco™ DMEM/F-12	Thermofisher	11,320,033
Gibco™ Neurobasal™ Medium	Thermofisher	21,103,049
Gibco™ StemPro™ Accutase™ Cell Dissociation Reagent	Thermofisher	A1110501
Glutamate	Sigma-Aldrich	G1626
GlutaMax	Thermo Scientific	35,050,087
HBSS	Gibco	14,065,049
Horse serum	BioConcept	2-05F00-1
K-HEPES	Research Organics Inc	6003H
KH_2_PO_4_	Sigma-Aldrich	P5655
L-Ascorbic acid	Sigma-Aldrich	A7506
Laminin	Roche Diagnostics	11,243,217,001
LDN193189	MedChem Express	HY-12071
Malate	Sigma-Aldrich	M1000
Mannitol	Sigma-Aldrich	M4125
MgCl_2_	Sigma-Aldrich	M4880
MitoSOX Red	Invitrogen	M36008
MTT	Merck	M2128
Neurobasal	Gibco	21,103–049
PBS	VWR	392–0440
Penicillin/Streptomycin	Bioconcept	4‐01F00‐5
PFA	Sigma-Aldrich	P6148
Poly-L-ornithine PLO	Sigma-Aldrich	P4957
Recombinant Human LIF	PeproTech	300–05
Recombinant Human CNTF	PeproTech	450–13
Retinoic acid	Sigma-Aldrich	R2625
Saccharose	Eurobio	018363
SB431542	LucernaChem	S-7800
Subtilisin A	MedChem Express	HY-E70076
Succinate	Sigma-Aldrich	S2378
TMRM	Fluka	87,919
TrypLE™ Select Enzyme (1X), no phenol red	Thermofisher	12,604–013
XAV939	LucernaChem	2,848,932
Y-27632 dihydrochloride, Rho kinase inhibitor (ab120129)	MedChem Express	HY-10583
Black 96 well plates	Greiner	655,090
White 96 well plates	Greiner	165,306
Clear 96 well plates	Falcon	353,072
Clear 12 well plate	Falcon	353,043
Collagen I precoated slide	Neuvitro	GC-18–1.5
Experimental models		
A172 untransfected	ATCC	CRL-1620
P301L-Tau-GFP transfected SH-SY5Y neuroblastoma cells (Human)	The Götz laboratory	
Healthy SH-SY5Y neuroblastoma cells transfected with GFP	ATCC	CRL-2266
Human induced pluripotent stem cells (iPSCs) from a patient bearing a P301Ltau mutation	Dr. Celeste Karch and the Tau Consortium Stem Cell Group	F0510.2 [20]
Human iPSCs from a heathy donor	Takara Bio	Cellartis® Human iPS Cell Line 12 (CHiPSC12)
Devices and software		
Eclipse Ti2 Nikon Microscope	Nikon	
Multiplate reader Cytation 3	Agilent	
Multiplate reader Cytation 5	Agilent	
Neurites Outgrowth Module	Agilent	
Resipher Lucid Lab	Lucid Scientific	https://lucidsci.com
Take3 Microvolume plate	Agilent	
Fiji (Fiji is just ImageJ) 2.16.0	NIH	https://imagej.net/software/fiji/
Prism version 10	GraphPad	https://www.graphpad.com/features
Huygens Deconvolution Software version 14.10.0	Scientific Volume Imaging	https://svi.nl/Huygens-Software
Imaris version 9.9.1	Oxford Instruments	https://imaris.oxinst.com

### Cell Lines

The human neuroblastoma cell line SH-SY5Y (ATCC, CRL-2266) was used as the recipient cells for the mitochondrial transplantation procedure. These cells have been documented in the scientific literature and are widely used as neuronal models in neuroscience research.

As a cellular model of tauopathy, we used human neuroblastoma SH-SY5Y cells stably overexpressing the P301L mutation (“P301L cells”) in the MAPT gene of chromosome 17 and tagged with a green fluorescent protein (GFP). This model was developed and generously provided by Professor Jürgen Götz's laboratory at the University of Queensland (Brisbane, Australia) [17]. The control cells consist of SH-SY5Y cells expressing the GFP-vector only ("Vector cells"). For both cell models (Vector and P301L cells), 4.5 µg/mL of blasticidin was added to the culture medium to maintain a stable expression of the plasmids.

Since intercellular mitochondria transfer naturally occurs between neurons and astrocytes, resulting in enhanced neuronal function and neuroprotection [18], the human astrocytic cell line A172 (ATCC, CRL-1620) was used as the mitochondria donor to provide healthy mitochondria for the bioenergetics experiments. In parallel, A172 cells stably expressing red fluorescent protein (RFP) or green fluorescent protein (GFP) tagged to mitochondria were used, allowing for the visualization of the transplanted astrocytic mitochondria in neuronal cells during microscopic experiments.

### Culture and Differentiation of SH-SY5Y Cells

SH-SY5Y cells were cultured in a growing medium composed of Dulbecco's Modified Eagle Medium (DMEM) supplemented with 1% penicillin and streptomycin, 1% glutamax, 10% fetal bovine serum (FBS), and 5% horse serum (HS). The selection antibiotic (blasticidin) was added during the medium change. The cells were cultivated in 10 cm^2^ culture dishes containing 10 mL of growing medium.

A172 cells were cultured in DMEM supplemented with 1% penicillin/streptomycin, 1% glutamax, and 10% FBS. These cells were maintained in 10 cm^2^ culture dishes and replated in a 20 cm^2^ dish before the mitochondrial isolation.

The cells were detached from the culture dishes using Accutase and split twice weekly once they reached approximately 80% confluency. The cells were subsequently stored in an incubator maintained at 37 °C and a CO_2_ concentration of 5%.

To be as close as possible to a neuronal phenotype, the SH-SY5Y Vector and P301L cells were differentiated into neuronal cells. Cells were plated in a 96-well plate format, and the growing medium was exchanged with 100 µL of differentiation medium 24 h after cell seeding. The differentiation medium comprised Neurobasal medium, 1% penicillin and streptomycin, 2% B-27, and 10 µM retinoic acid. A washing step with phosphate-buffered saline (PBS) was performed before the incubation with the differentiation medium to ensure the complete removal of FBS and HS. The cells were maintained in the differentiation medium for three days. Subsequently, half of the volume of the differentiation medium was replaced to refresh the medium before the mitochondrial transplantation.

### Culture and Differentiation of iPSC-Derived Neurons and Astrocytes

Human mutant (MAPT P301L/WT) induced pluripotent stem cells (iPSCs) were kindly provided by Dr. Celeste Karch and the Tau Consortium Stem Cell Group [20]. The iPSCs derived from a healthy donor were purchased from Takara Bio (Kusatsu, Shiga, Japan). Essential 8™ (E8) Medium containing Essential 8™ Supplement and 1% of Penicillin/Streptomycin (P/S). The iPSCs were kept in a humidified incubator at 37 °C and 5% CO_2_. The E8 medium was replaced every day and cells were passaged twice a week using TrypLE Select. During passaging or plating, 20 μM of Rock-inhibitor Y-27632 was added to the E8 medium to minimize cell death.

Neural induction of iPSCs to neural progenitor cells (NPCs) was conducted in three steps. First, cells were cultured on Geltrex-coated plates in Neural Induction Medium I (NIM I) composed of Advanced DMEM:F12 base medium with 1% P/S, 2 mM Glutamax, 1% B27 supplement, 10 μM SB-431542, 1 μM LDN-193189, and 2 μM XAV939. The medium was changed daily, and cells were passaged when they reached confluency using TrypLE Select. Then, after 7 days, NIM I was replaced by Neural Induction Medium II (NIM II) composed of Advanced DMEM:F12 base medium with 1% P/S, 2 mM Glutamax, 1% B27 supplement, 200 nM LDN-193189, and 2 μM XAV939. Again, the medium was changed daily, and cells were passaged when they reached confluency using TrypLE Select. Finally, after 7 days, NIM II was replaced by Neural Induction Medium III (NIM III) composed of Advanced DMEM:F12 base medium with 1% P/S, 2 mM Glutamax, 1% B27 supplement, and 20 μg/ml FGF-2 (147). This step allowed the NPCs to proliferate, and a Master Stock was constituted for the experiments. The quality of NPCs was controlled using the Human Neural Stem Cell Immunocytochemistry Kit to verify whether the markers of human neural stem cells SOX 1, SOX 2, Nestin, and PAX6 are well expressed (Supplementary Fig. 1a, b). Expanded NPCs were cryopreserved in a freezing medium composed of Advanced DMEM:F12 base medium, 1% P/S, 2 mM Glutamax, 10% DMSO, and 20 μM of Rock-inhibitor Y-27632, and kept in a liquid nitrogen tank until use.

For the neuronal differentiation, P301L mutant NPCs were thawed and plated on Geltrex-coated plates in NIM III. Once they recovered from thawing, NIM III was replaced with the Neuronal Differentiation medium I (ND I) composed of Neurobasal Medium with 1% P/S, 2 mM Glutamax, 1% B27 supplement, and 5 μM DAPT. Once the cells stopped proliferating, they were plated on PLO/laminin-coated plates at 40.000 cells/cm^2^ density. After 7 days in ND I, the medium was replaced with Neuronal Differentiation medium II (ND II) composed of Neurobasal Medium with 1% P/S, 2 mM Glutamax, 1% B27 supplement, and 5 μM DAPT, 200 μM L-ascorbic acid, 1.8 mM CaCl_2_, and 20 ng/ml BDNF, and changed twice per week. After 14 days of differentiation, neurons were plated in 96-well plates at a density of 20.000 cells/well. Immunostainings were performed after 28 days of differentiation to confirm neuronal marker expression B3TUB and MAP2 (Supplementary Fig. 1c). After 28 days of differentiation from the NPC stage, the neurons were used for the transplantation experiments.

CHiPSC12-derived NPCs were differentiated into astrocytes as previously described [21]. NPCs were thawed and plated on Geltrex-coated plates in NIM III. Once they recovered from thawing, NIM III was replaced with the Astrocyte Induction Medium (AIM) composed of DMEM:F12 base medium with 1% P/S, 2 mM Glutamax, 1% B27 supplement, 1% N2 supplement, 10 ng/ml EGF, and 10 ng/ml LIF. The AIM was changed every other day for 14 days. Cells were passaged when they reached confluency using TrypLE Select, and plated at a density of 50.000 cells/cm^2^. On day 14, AIM was replaced with the Astrocyte Maturation Medium (AMM) composed of DMEM:F12 base medium with 1% P/S, 2 mM Glutamax, 1% B27 supplement, and 20 ng/ml CNTF. Medium was changed every other day and cells were passaged when they reached confluency using TrypLE Select, and plated at a density of 30.000–40.000 cells/cm^2^. Immunostainings were performed on day 42 of differentiation to confirm astrocytic marker expression GFAP, GLAST, and S100B (Supplementary Fig.  1d). As described in the original protocol, more than 95% of the iPSC-derived astrocytes co-expressed GLAST and S100B [21]. A variable proportion of the cells expressed GFAP. Expanded astrocytes were cryopreserved in a freezing medium composed of DMEM:F12 base medium, 1% P/S, 2 mM Glutamax, 10% DMSO, and 20 μM of Rock-inhibitor Y-27632, and kept in a liquid nitrogen tank until use. Astrocytes were thawed in a 10 cm^2^ culture dish in AMM containing 10 μM of Rock-inhibitor Y-27632. Y-27632 was removed one day after thawing. Cells were then replated in a 15 cm^2^ dish at a density of 4.5 × 10^6 cells per dish and used for mitochondrial isolation one week after thawing (D49 post-NPC stage).

### Mitochondrial Isolation

The protocol for mitochondrial isolation has been previously established by Preble et al. in 2014 [22]. This filtration-based mitochondria isolation protocol enables the isolation of viable mitochondria with less than 0.001% contamination by non-mitochondrial particles. Importantly, this method is patented (WO2015192020A1) and already used in a clinical context [23]. All procedures were conducted in a controlled, sterile environment. For each mitochondria isolation, a full dish of 20 cm^2^ of A172 cells was used. A homogenization buffer was prepared, composed of 300 mM saccharose, 10 mM K-HEPES, and 1 mM EGTA, which were diluted in ultrapure water. The pH was adjusted to 7.2 with a 1 M NaOH solution. The solution was filtered under sterile conditions using a 0.2 µm filter and stored at 4 °C until use.

Medium was removed from the dish containing A172 cells, followed by one wash with PBS. Then, 1 mL of homogenization buffer was added to the dish, and the cells were mechanically harvested using a cell scraper. The cells were centrifuged at 4 °C for 5 min at 700 × g. Next, the cell pellet was suspended in 2 mL of homogenization buffer, and cells were lysed using a Potter grinder. A solution containing 4 mg/ml Subtilisin A was added to the tube and incubated on ice for 10 min. Filtration steps were then performed to remove the majority of cell debris. First, the cell lysate was filtered using a 40 µm mesh size filter. Then, 500 µL of 20 mg/ml BSA (bovine serum albumin) was added to the lysate before a second filtration step with a new filter with a 40 µm mesh size, followed by a third filtration with a mesh size of 10 µm. Finally, the filtered cell lysate was centrifuged at 4 °C for 10 min at 10,000 × g. The final pellet was suspended in 200 µL of mitochondrial assay solution (MAS) and stored on ice until the transplantation step was performed. Of note, the MAS is composed of 200 mM mannitol, 70 mM saccharose, 10 mM KH_2_PO_4_, 5 mM MgCl_2_, 2 mM K-HEPES, 1 mM K-EGTA, and 0.2% BSA in 200 mL of ultrapure water [24]. The MAS pH is adjusted to 7.2, and the solution is frozen at −20 °C for extended periods.

### Estimation of Mitochondrial Concentration

The concentration of isolated mitochondria was determined by conducting a protein assay using the Cytation 3 Cell Imaging Multi-mode Plate Reader (BioTek, Agilent, Switzerland) and the Take3 Microvolume plate. The software utilized for data analysis was Gen3.16. The protein absorption was measured at a wavelength of 280 nm (nm), and the MAS was used for the blank measurement. This approach is reliable, efficient, and fast, thereby ensuring optimal conditions for preserving the integrity of isolated mitochondria before transplantation.

### Mitochondrial Transplantation

Isolated mitochondria were kept on ice in the MAS for less than 1 h. The acceptor cells (SH-SY5Y cells) were incubated with the isolated mitochondria (mitochondria-transplanted group), while the MAS alone was used as the control condition. A final volume of 10 µL of isolated mitochondria was added to the 100 µL of medium in a 96-well plate containing neuroblastoma cells. The final concentrations of isolated mitochondria tested were 5 µg/mL, 10 µg/mL, 25 µg/mL, and 50 µg/mL. The selection of these concentrations was guided by the findings from Shi et al., who conducted a study to identify the most effective concentrations for SH-SY5Y cells [14]. The cell plate was then incubated at 37 °C and 5% CO_2_ for 24 or 48 h.

### Validation of the Entry of Isolated Mitochondria Into Recipient Cells

Cells were plated in a 12-well plate on collagen I-precoated coverslips at a density of 5 × 10^^4^ cells per well and differentiated using the previously described differentiation protocol. To investigate whether isolated mitochondria can enter cells, A172 cells expressing the RFP-tagged mitochondria (mitoRFP) were used as a mitochondrial source. After 24 and 48 h of co-incubation with isolated mitochondria, SH-SY5Y cells were either incubated with 5 µM of CellTracker Blue dye for 30 min at 37 °C to visualize the cells’ area. Then, cells were washed with PBS and fixed with 4% paraformaldehyde (PFA) for 10 min at room temperature (RT). Pictures were captured using an Eclipse Ti2 widefield microscope (Nikon). Images were subjected to a deconvolution step using the Huygens Deconvolution Software. Subsequent analysis was conducted using the Fiji and Imaris softwares.

### Estimation of Mitochondria Entry Into Recipient Cells

Cells were plated in a collagen-coated black 96-well plate with a clear bottom at a density of 5 × 10^^3^ cells/well. One day after plating, the cells were placed in medium containing Neurobasal, 1% penicillin, 1% streptomycin, 2% B27, and 10 µM retinoic acid. After three days, the cells were treated with 25 µg/mL of mitochondria isolated from mito-GFP-expressing A172 cells. After 24 and 48 h, cells were fixed using 4% paraformaldehyde (PFA), permeabilized with 0.2% Triton X-100 in PBS, and blocked with 2% BSA. Immunostaining was then performed to visualize the cells’ area using an anti-ß3 tubulin mouse primary antibody (1:1000) and an anti-mouse secondary antibody coupled with Alexa Fluor 568 (1:1000). Nuclear staining was performed by adding DAPI during the last wash step. Cells were then placed in 100 µL of PBS and kept at 4 °C until use.

Fluorescence microscopy was performed using the Cytation 5 Imaging Multi-Mode Reader (BioTek, Agilent, Switzerland) with Gen5 software. Pictures were taken using the DAPI filter (to visualize the nuclei), the GFP filter (to visualize the mitoGFP transplanted mitochondria), and the RFP filter (to visualize the ß3 tubulin staining, and therefore the SH-SY5Y cells’ area). Image acquisition was performed using a 20 × PL FL objective. Autofocus and auto-exposure functions were applied to all channels, and pictures were taken automatically. Namely, 10–15 images were taken per well, with at least three wells per condition. Batch cell analysis was then performed using the Gen5 software on the Cytation 5 microplate reader. A primary mask was created based on the red fluorescence signal (RFP) to define the area of individual cells. A secondary mask was then generated within the primary mask to detect the GFP signal within the RFP area. The software then gives the proportion of mitoGFP-positive cells in the total cell population. The analysis protocol was standardized across all experimental conditions to ensure reproducibility. This microscopy experiment was repeated at least three times.

### Assessment of Isolated Mitochondria Integrity

To assess the mitochondrial integrity after isolation, a cytochrome c permeability assay was performed using the Seahorse HS Mini (Agilent). After isolation of mitoGFP mitochondria from A172 cells and protein quantification, mitochondria were diluted at a final concentration of 100 µg/mL in MAS containing 10 mM sodium pyruvate, 10 mM succinate, and 2 mM malate. In a Seahorse Mini cell plate, 50 µL of mitochondria in MAS + substrates were placed, and centrifuged at 2000 g for 20 min at 4 °C. Then, 150 µL of pre-warmed (37 °C) MAS + substrates were added to each well, and the plate was placed in the Seahorse HS Mini. After measuring the basal oxygen consumption rate (OCR), adenosine diphosphate (ADP, 2 mM) was injected to induce respiratory state 3. Then, cytochrome c (10 µM) was injected to assess mitochondrial membrane integrity. Finally, rotenone (1 µM) and antimycin A (1 µM) were injected to inhibit electron transport chain activity. At the end of the measurement, the Seahorse plate containing mitochondria was placed in the Cytation 5 Imaging Multi-Mode Reader, and the fluorescence intensity of the mitoGFP signal was measured at an excitation wavelength of 480 nm and an emission wavelength of 520 nm. The Seahorse data were uploaded to the Seahorse Analytics software and normalized on the mitoGFP signal.

### Normalization of Bioenergetic Assays

Data normalization was performed using the CellTracker Blue CMAC fluorescent signal. After loading into living cells, the dye is retained, and the fluorescent emission represents the cell density in the well. The cells were incubated with 5 µM of CellTracker Blue for 30 min at 37 °C. Two washing steps were then performed with HBSS and the fluorescence intensity (Excitation/Emission: 353/466 nm) was detected with the Cytation 3 Cell Imaging Multi-mode Plate Reader (BioTek, Agilent, Switzerland). This normalization step was realized for the ATP assay, mitochondrial membrane potential determination, oxygen consumption rate measurement and superoxide anion radical quantification.

### Cellular Viability Assay

The cellular viability was measured using the MTT (3-(4,5-dimethylthiazol-2-yl)−2,5-diphenyltetrazolium bromide) assay. This colorimetric test reflects the metabolic activity of living cells and is interpreted as an indicator of cellular viability and toxicity. The experiment was conducted by seeding 1.5 × 10^4^ SH-SY5Y cells per well in clear 96-well plates. The MTT was first diluted in culture medium to 5 mg/mL. Then, the diluted MTT was added to the SH-SY5Y cells and incubated for 2 h at 37 °C and 5% CO_2_. The medium was carefully removed from the wells and substituted with 150 µL of dimethyl sulfoxide (DMSO). The plate was then placed on a shaker at 250 rpm for 15 min. The resulting absorption was measured at a wavelength of 550 nm using the Cytation 3 instrument. This test was conducted 24 h and 48 h after the mitochondrial transplantation.

### ATP levels

The test was conducted using the "ATPlite 1-step Luminescence Assay System" kit. The assay is based on a bioluminescence reaction between an enzyme, luciferase, and a substrate, luciferin. This reaction requires the presence of ATP, with the emitted light being proportional to the level of ATP in each well. The cells were seeded in white 96-well plates (1.5 × 10^^4^ cells/well) to optimize the signal in the plate reader. An ATP standard (ranging from 0 to 20 µM) was prepared in duplicate to calculate the ATP concentration in our samples. To initiate the enzymatic reaction, 100 µL of ATP substrate was added to each well. Then, the plate was placed on the shaker at 200 rpm for 2 min, and the bioluminescence signal was read with the Cytation 3 instrument. The tests were conducted at 24 h and 48 h post-transplantation.

### Determination of Mitochondrial Membrane Potential

The mitochondrial membrane potential (MMP) was determined using the TMRM dye (tetramethylrhodamine methyl ester). The dye’s capacity to enter the mitochondrial matrix is proportional to the MMP.

To assess the membrane potential of mitochondria freshly isolated from A172 cells and confirm their bioenergetic activity and viability, two tubes containing 100 µg/mL of isolated mitochondria in MAS were prepared and incubated with 0.4 µM TMRM for 30 min at 37 °C. Subsequently, the samples were centrifuged at 10,000 × g for 10 min at room temperature. Then, the mitochondria pellet from the first tube was resuspended in the MAS. The second pellet was resuspended in the MAS supplemented with 0.05 mM Glutamate, 8 mM Succinate, and 5 mM Malate, which are substrates required for the electron transport chain activity. Finally, 50 µL of samples were added to black 96-well plates, and the emitted fluorescence (Excitation/Emission: 550/580 nm) was detected using the Cytation 3.

To assess the effects of mitochondrial transplantation on the MMP of neuronal cells, SH-SY5Y cells were seeded in black 96-well plates at a density of 1.5 × 10^^4^ cells/well. The measurement of the MMP was conducted at 24 h and 48 h following transplantation with isolated mitochondria. The TMRM dye was added to each well at a final concentration of 0.4 µM. The plate was then placed in the dark on a shaker at 100 rpm for 30 min. Two washing steps were then performed with HBSS, and the fluorescence intensity (Excitation/Emission: 550/580 nm) was measured with the Cytation 3.

### Assessment of Superoxide Anion Radical Levels

The total and mitochondrial superoxide anion levels were measured using the DHE (dihydroethidium) and MitoSOX Red dyes, respectively. The cells were plated in black 96-well plates at a density of 1.5 × 10^^4^ cells/well. The superoxide anion levels were determined at 24 h and 48 h post-transplantation. The cells were incubated with 10 µM DHE for 20 min in the dark at room temperature or with 50 µM MitoSOX red for 90 min at 37 °C and 5% CO_2_. Cells were then washed twice with HBSS, and the fluorescence intensity (Excitation/Emission: 531/595 nm), which is proportional to the total or mitochondrial superoxide anion levels, was detected with the Cytation 3.

### Cellular Respiration Measurement

The Resipher device was used to assess cellular respiration over time. This device enables the direct measurement of oxygen consumption rate (OCR) in real-time within the cell incubator (Lucid Scientific [25]). The Resipher device was affixed magnetically to a lid containing sensors placed on 96-well plates with 1.5 × 10^^4^ SH-SY5Y cells/well. The medium volume of 90 µL per well was selected based on the recommendations of Lucid lab to ensure optimal measurement conditions. The experimental protocol started 24 h prior to transplantation and ended 48 h post-transplantation. The OCR values were extracted on the Lucid Scientific platform.

### BrdU Cell Proliferation Assay

To ascertain that the effect observed after the mitochondrial transplantation was not attributable to a proliferative effect of undifferentiated SH-SY5Y cells, a BrdU cell proliferation assay (Merck, QIA58) was used. This assay is a non-isotopic enzymatic immunoassay quantifying BrdU incorporation into newly synthesized DNA of actively proliferating cells. In brief, BrdU (1:2000) was incubated with undifferentiated SH-SY5Y cells overnight to allow at least one complete cell division cycle. The following day, the sample underwent fixation and denaturation, followed by anti-BrdU immunostaining. The anti-BrdU secondary antibody is coupled with the horseradish peroxidase (HRP) enzyme, producing a colored product. The reaction is then stopped using a stop solution provided in the kit. The absorbances were measured at 450 nm and 540 nm (reference) using the Cytation 3. The final values, corresponding to the difference between the absorbances at 450 nm and 540 nm, are proportional to cell proliferation.

### Neurite Outgrowth Measurement

The impact of mitochondrial transplantation on neuronal plasticity was assessed using the Agilent BioTek Gen5 Neurite Outgrowth Module. The experiments were conducted in a blinded manner since we used an automated module applying pre-defined parameters to all sample batches. Cells were seeded in black 96-well plates at a density of 5 × 10^^3^ cells/well and maintained in the differentiation medium for three days. The differentiation medium comprised Neurobasal, 1% penicillin, 1% streptomycin, 2% B27, and 10 µM retinoic acid. After three days, the differentiation medium was replaced with Neurobasal medium containing 1% penicillin and streptomycin, 2% B27, and different treatment conditions: MAS only (control), 25 µg/mL of isolated mitochondria, or 50 ng/mL nerve growth factor (NGF) as a positive control.

After 48 h of treatment, immunostaining with anti-ß3 tubulin was performed. Cells were fixed with 4% PFA for 10 min at room temperature (RT) and permeabilized with 0.15% Triton X-100 in PBS for 15 min. Samples were incubated with 2% BSA in PBS to block unspecific sites for 1 h. The primary antibody (anti-ß3 tubulin, chicken, 1:1000) was incubated overnight at 4 °C. After three washes with PBS, the secondary antibody (anti-chicken and preabsorbed with Alexa Fluor 488, 1:500) was subsequently incubated for 1 h in the dark at RT. Finally, the cells were incubated with a nuclear staining solution (DAPI, 1:1000) for 5 min at RT. The samples were then kept in PBS and stored at 4 °C.

Data from the neurite outgrowth experiments were obtained with the Cytation 5 Cell Imaging Multi-mode Plate Reader (BioTek, Agilent, Switzerland). Pictures were submitted to a preprocessing step with the predefined parameters in the software for both channel DAPI and GFP (rolling ball diameter: 138 µm). Then, a deconvolution step was applied to the preprocessed images (Point spread function: “Auto, based on objective”; number of iterations: 5). The Neurite Outgrowth Module was used on the deconvolved images with experimental settings established for Vector and P301L cells. The parameters collected for this study were: the average neurite length (the total skeleton length of all neurites divided by the soma count), the average neurite count per cell (number of neurites connected to the identified soma), average neurites branches per cell (the number of ending branches connecting to neurites divided by the soma count) and the neurite thickness (corresponding to the total neurite area divided by the total neurite length, in µm).

### Statistical Analysis

All results were exported to Microsoft Excel 2019® and analyzed using GraphPad Prism 10® software. The results, depicted as boxes and whisker plots, indicate the median value, the mean, and the distribution of values with the minimal and maximal points ("whiskers"). The values are expressed as percentages of the control (MAS) condition. To ensure the reliability and reproducibility of the experimental results, each experiment was performed at least three times.

The statistical analysis employed was One-way ANOVA with a post-hoc Dunnett's multiple comparison test versus the control condition or Two-ways ANOVA with a post-hoc Tuckey's multiple comparison test. Results were considered statistically significant if p-values < 0.05.

## Results

### Establishment of the optimal parameters for mitochondrial transplantation in the SH-SY5Y cells

**Fig. 1:**
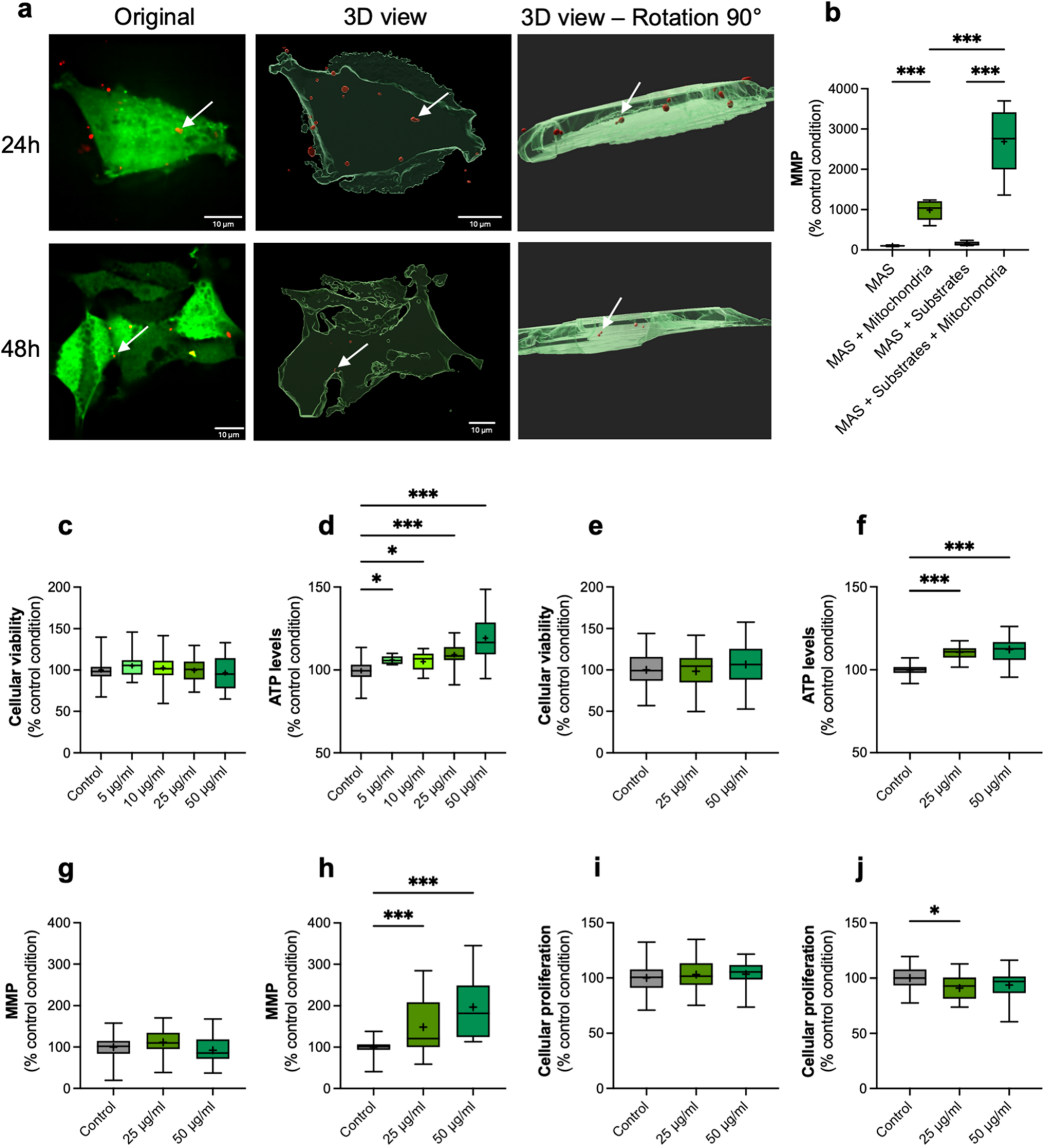
Mitochondrial transplantation in undifferentiated SH-SY5Y cells. **(a)** SH-SY5Y cells stained with the CellTracker blue dye (green false color), and mitochondria isolated from A172 cells expressing the mitoRFP tag are shown in red. Images were captured at 100 × magnification with the fluorescent Eclipse Nikon Microscope after 24 and 48 h of incubation with isolated mitochondria. 3D representations were obtained with the Imaris software; the white arrows shows to transplanted mitochondria into the recipient cells. (**b**) Determination of the mitochondrial membrane potential (MMP) of isolated mitochondria. The MMP of isolated mitochondria was measured on mitochondria in MAS (mitochondria assay solution), or mitochondrial in MAS + substrates (glutamate, succinate, and malate) to stimulate the electron transport chain activity) (**c**,** e**) Estimation of cellular viability at 24 h (**c**) and 48 h (**e**) post-transplantation with 5 µg/mL, 10 µg/mL, 25 µg/mL, and 50 µg/mL of isolated mitochondria. (**d**,** f**) Relative quantification of ATP levels at 24 h (**d**) and 48 h after normalization on the CellTracker Blue signal. (**f**). (**g-h**) Determination of the MMP at 24 h (**g**) and 48 h (**h**) after mitochondrial transplantation and normalization on the CellTracker Blue signal. (**i-j**) Cellular proliferation effects of mitochondrial transplantation at 24 h (**i**) and 48 h (**j**). The boxes represent the median (full line) and the mean (“ + ” symbol), and the whiskers represent the minimal and maximal values. Each data set represents *N* = 3 independent experiments with 7–10 replicates per condition (20–30 total replicate number per condition). Values are shown as the percentage of the control condition. One-way ANOVA and post hoc Dunnett's multiple comparison test versus the control condition. *p < 0,05; ***p < 0,001. ATP: adenosine triphosphate, MAS: mitochondrial assay solution, MMP: mitochondrial membrane potential

Astrocytic mitochondria were freshly isolated from the A172 cells. Microscopic experiments were performed to assess the ability of isolated mitochondria to enter recipient cells. As shown in the microscopy pictures and 3D representations in Fig. 1a, isolated mitochondria are present in the recipient cells after 24 h and 48 h of incubation (see also Supplementary Movies 1 to 4). Microscopy analysis revealed that, on average, 26–27% of SH-SY5Y cells were positive for A172-derived mitochondria (Supplementary Fig. 2a, b). There was no statistical difference between the 24- and 48-h timepoints (Supplementary Fig. 2b).

To ensure that the isolated mitochondria remained healthy and functional, we first assessed whether their mitochondrial membrane potential (MMP) was preserved after isolation and whether the electron transport chain (ETC) activity remained efficient. We observed that the TMRM dye was still capable of entering isolated mitochondria and emitting a fluorescent signal, which reflected a polarized membrane potential (Fig. 1b). The addition of ETC substrates (glutamate, succinate, and malate) resulted in an increase in MMP compared to the control without substrates (*p* < 0.001). To verify mitochondrial integrity after isolation, we measured the oxygen consumption rate (OCR) of isolated mitochondria (Supplementary Fig. 2c). We observed that OCR increased after the addition of adenosine diphosphate (ADP), indicating that mitochondria can enter respiratory state 3. However, no change in OCR was observed after the addition of cytochrome c, suggesting that the mitochondrial membrane remained intact. Together, these results showed that the functional integrity of mitochondria was preserved after the isolation process.

The next step was to determine the optimal concentration and incubation time to observe an effect after transplantation of isolated mitochondria. Initial trials were conducted on healthy and undifferentiated SH-SY5Y cells. Based on the methodologies outlined by Shi et al. [14], a range of concentrations was selected for subsequent experimentation: 5, 10, 25, and 50 µg/mL, and two time points: 24 h and 48 h.

First, we assessed whether the treatment with isolated mitochondria was toxic to cells. To do so, we performed an MTT viability assay. No significant difference was observed between the control (no mitochondria) and all the tested conditions (from 5 µg/mL to 50 µg/mL) at 24 h after mitochondrial transplantation (Fig. 1c). Although mitochondrial transplantation did not increase cell viability, it was not toxic for the cells. In parallel, we assessed the effect of the treatment on cell bioenergetic activity using an ATP assay. The same experimental conditions were used as previously for the MTT assay. A dose–response curve was observed after one day of transplantation, with a significant increase for the treated conditions compared to the control group (Fig. 1d). Especially, a + 9% and + 19% increase in ATP levels were observed with the 25 and 50 µg/mL, respectively (*p* < 0.001). These two treatment concentrations were selected to continue the screening phase at 48 h post-transplantation. We confirmed the MTT and ATP results 48 h after transplantation. As observed on the graphs (Fig. 1e, f), the same trend was observed after two days of treatment, with no difference between the control and treated conditions regarding the cellular viability (Fig. 1e) but an increase in cellular metabolic activity (+ 10% and + 12% increase for the 25 and 50 µg/mL conditions, respectively) (*p* < 0.001, Fig. 1f).

Since it is known that ATP production is closely related to the mitochondrial membrane potential (MMP), this parameter was measured using the TMRM dye. In comparison with the control group, no difference was observed after 24 h of treatment (Fig. 1g). A significant increase in MMP was detected after 2 days of incubation for both treated conditions compared to the control (Fig. 1h). At this time point, MMP is statistically higher in the 50 µg/mL condition (+ 96% increase versus the control) than in the 25 µg/mL condition (+ 48% increase versus the control) (*p* < 0.001). A BrdU assay was performed to ensure that these beneficial effects were not due to an increase in cell proliferation after treatment. No variation in cell proliferation was observed at 24 h post-transplantation for both tested concentrations (Fig. 1i). In addition, a decrease in this parameter (- 9%) was observed after treatment with 25 µg/mL of isolated mitochondria for 48 h (*p* = 0.014, Fig. 1j). Therefore, all the beneficial effects observed in the previous data were due to mitochondrial transplantation and not to cell proliferation.

Based on all these results, the 25 µg/mL mitochondria concentration was selected for subsequent analysis. Indeed, this concentration showed a beneficial effect on cell functions with no obvious difference with the 50 µg/mL treatment condition.

### Mitochondrial transplantation enhances bioenergetic functions and mitigates oxidative stress in the Vector SH-SY5Y cells

To approach a neuronal model more closely, the second part of the experiments was performed on differentiated and healthy SH-SY5Y cells ("Vector" cells). Based on the results from the screening phase, the optimal treatment concentration was determined to be 25 µg/mL. The same bioenergetic assays were performed on the differentiated Vector cells, with additional assessments of superoxide anion radical levels and cellular respiration at 24 h and 48 h post-transplantation.

**Fig. 2:**
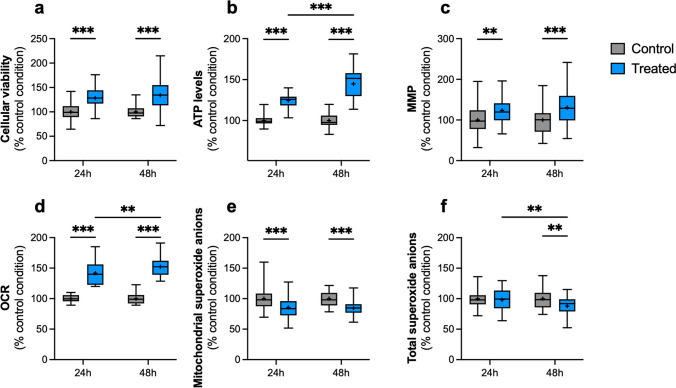
Impact of mitochondrial transplantation on the cellular viability and bioenergetic parameters in differentiated Vector SH-SY5Y cells. The « control» condition corresponds to the vehicle-treated condition with the mitochondrial assay solution. The « mitochondria» condition corresponds to the treatment with 25 µg/mL of isolated mitochondria. (**a**) Estimation of cellular viability at 24 h and 48 h post-transplantation. (**b**) Relative quantification of ATP levels at 24 h and 48 h post-transplantation. (**c**) Determination of MMP at 24 h and 48 h post-transplantation. (**d**) Measurement of OCR at 24 h and 48 h post-transplantation. (**e**) Relative levels of mitochondrial superoxide anions at 24 h and 48 h post-transplantation. (**f**) Relative quantification of the total superoxide anions at 24 h and 48 h post-transplantation (**g**). Each experiment was normalized on the corresponding CellTracker Blue fluorescent signal. The boxes represent the median (full line) and the mean (“ + ” symbol), the whiskers represent the minimal and maximal values. Each data set represents *N* = 3 independent experiments with 10–15 replicates per condition (30–40 total replicate number per condition). Values are shown as the percentage of the control condition. Two-way ANOVA and post hoc Tukey’s multiple comparison tests.; **p < 0,01; ***p < 0,001. ATP: adenosine triphosphate, MMP: mitochondrial membrane potential, OCR: oxygen consumption rate

The potential toxicity of the treatment was assessed at 24 h and 48 h post-transplantation. We observed an increase in viability compared to the corresponding control condition of + 28% and + 34%, respectively (*p* < 0.001). However, no difference was observed between the treated conditions at 24 h and 48 h (*p* = 0.068, Fig. 2a). An increase in cellular metabolism was also observed, with a 25% and 45% increase in ATP production after treatment at 24 h and 48 h, respectively (*p* < 0.001). For this parameter, the level of ATP is significantly higher at 48 h post-transplantation compared to the treated group at 24 h (*p* < 0.001, Fig. 2b). In parallel, MMP was increased by + 24% and + 31% after one and two days of treatment, respectively, showing a correlation with ATP level (*p* = 0.003 and *p* < 0.001 respectively). However, there was no difference between the treated condition at 24 h and 48 h after transplantation (*p* = 0.332, Fig. 2c).

We next assessed whether the treatment's effects were related to an increase in cellular respiration by measuring the oxygen consumption rate (OCR) in the recipient cells. Compared to the corresponding control condition, an increase in OCR was observed at 24 h (+ 42%) and 48 h (+ 52%) after transplantation (*p* < 0.001). This increase appeared stable over time, as there was no significant difference between the treated conditions at both time points (*p* = 0.004, Fig. 2d).

The primary risk associated with enhancing the bioenergetic functions of cells is the concurrent increase in reactive oxygen species (ROS) production. Therefore, cellular and mitochondrial superoxide anion radical levels were measured using fluorescent dyes. Surprisingly, mitochondrial superoxide anion levels decreased at 24 h (−14%) and 48 h (−16%) after treatment (*p* < 0.001). No difference was observed between the two time-points (Fig. 2e), indicating that this effect is maintained over time. In addition, total superoxide anion radical levels were decreased at 48 h (−12%) after transplantation with isolated mitochondria (*p* = 0.003, Fig. 2f).

Thus, mitochondrial transplantation improves the cellular viability and bioenergetic functions of differentiated Vector cells. Additionally, a decrease in oxidative stress was observed following treatment. Most of the results were similar at 24 h and 48 h post-transplantation, indicating that the transplantation may have a sustainable positive effect.

### Mitochondrial Transplantation Enhances the Bioenergetics Functions and Reduces the Mitochondrial Superoxide Anion Levels in P301L SH-SY5Y Cells

Based on the data obtained in the Vector cells, we next performed the same experiments in P301L SH-SY5Y cells at the same time points, 24 h and 48 h. These cells are known to present bioenergetic deficits [26], therefore we aimed to assess whether mitochondrial transplantation could also enhance cell metabolism in a pathological model.

**Fig. 3:**
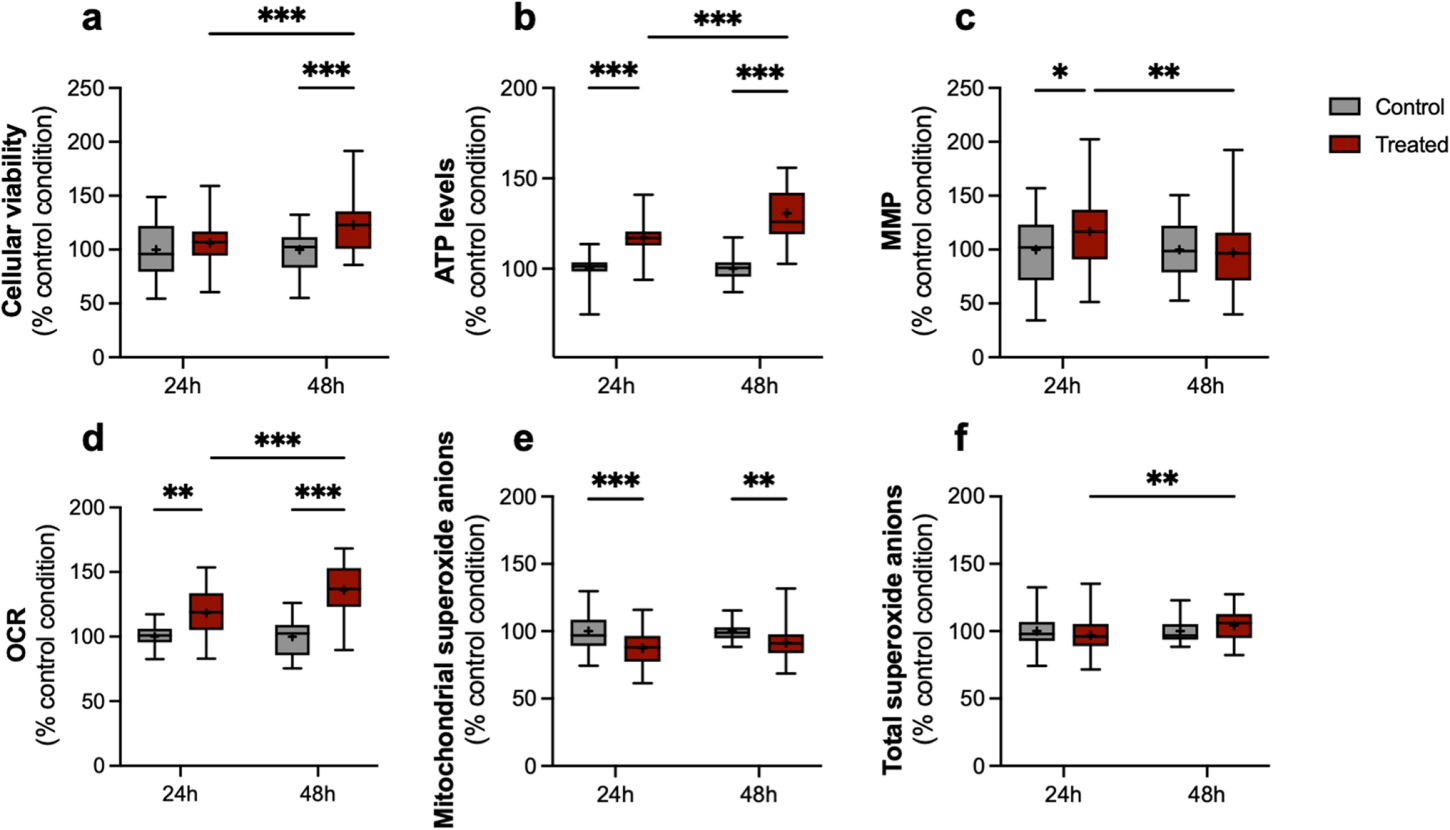
Impact of mitochondrial transplantation on the cellular viability and the bioenergetic features in differentiated P301L SH-SY5Y cells. The « control» condition corresponds to the vehicle-treated condition with the mitochondrial assay solution. The « mitochondria» condition corresponds to the treatment with 25 µg/mL of isolated mitochondria. (**a**) Estimation of cellular viability at 24 h and 48 h post-transplantation. (**b**) Relative quantification of ATP levels at 24 h and 48 h post-transplantation. (**c**) Determination of MMP at 24 h and 48 h post-transplantation. (**d**) Measurement of OCR at 24 h and 48 h post-transplantation. (**e**) Relative levels of mitochondrial superoxide anions at 24 h and 48 h post-transplantation. (**f**) Relative quantification of the total superoxide anions at 24 h and 48 h post-transplantation (**g**). Each experiment was normalized on the corresponding CellTracker Blue fluorescent signal. The boxes represent the median (full line) and the mean (“ + ” symbol), the whiskers represent the minimal and maximal values. Each data set represents *N* = 3 independent experiments with 10–15 replicates per condition (30–40 total replicate number per condition). Values are shown as the percentage of the control condition. Two-way ANOVA and post hoc Tukey’s multiple comparison tests. *p < 0,05; **p < 0,01; ***p < 0,001. ATP: adenosine triphosphate, MMP: mitochondrial membrane potential, OCR: oxygen consumption rate

Cell viability of P301L cells was improved at 48 h post-transplantation with an increase of + 22% compared to the untreated condition (*p* < 0.001). No significant differences were observed at 24 h (Fig. 3a). In parallel, an increase in ATP level was observed at 24 h (+ 17%) and 48 h (+ 31%) compared to the control condition (*p* < 0.001). The effect of the treatment was higher at 48 h than 24 h (*p* < 0.001, Fig. 3b). The MMP was significantly increased in P301L cells 24 h post-transplantation (*p* = 0.025). However, the effect was lost after 48 h (Fig. 3c). Cellular respiration was also assessed in P301L cells. An increased OCR was observed after treatment at 24 h (+ 18%) and at 48 h (+ 36%) compared to the corresponding control condition (*p* = 0.02 and *p* < 0.001 respectively, Fig. 3d). As for the ATP production, the respiratory capacity of the cells seemed to correlate with the incubation time of the isolated mitochondria with the P301L cells.

A previous study of our group has shown that the ROS level is higher in the P301L cells than in the Vector cells [26]. Thus, the impact of mitochondrial transplantation on oxidative stress was an essential point to assess in this model. Therefore, total and mitochondrial superoxide anion radical levels were measured in P301L cells after mitochondria transplantation. We observed a significant decrease in mitochondrial ROS levels at 24 h (−13%) and at 48 h (−9%) compared to the corresponding control condition (*p* < 0.001 and *p* = 0.006 respectively, Fig. 3e). This effect remained stable over time, with no statistical difference between the treated condition at either time point. However, no decrease in the total ROS levels was observed after treatment (Fig. 3f).

Overall, mitochondrial transplantation enhanced the cellular viability and bioenergetic functions of P301L cells after 24 h and 48 h of incubation. Additionally, mitochondrial oxidative stress was reduced following treatment.

### Mitochondrial Transplantation Enhances the Bioenergetics Functions in P301L Mutant IPSC-derived Neurons

**Fig. 4:**
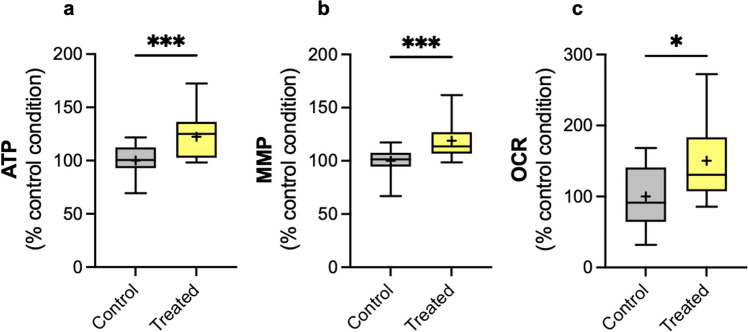
Mitochondrial transplantation increases bioenergetic features in iPSC-derived neurons bearing the P301L mutation. The « control» condition corresponds to the vehicle-treated condition with the mitochondrial assay solution. The « treated» condition corresponds to the treatment with 25 µg/mL of mitochondria isolated from iPSC-derived astrocytes. (**a**) Quantification of ATP levels, (**b**) MMP, and (**c**) OCR in P301L mutant iPSC-derived neurons 48 h post-transplantation. The boxes represent the median (full line) and the mean (“ + ” symbol), the whiskers represent the minimal and maximal values. Each graph represents data from *N* = 3 independent experiments with 10–15 replicates per condition (32–40 total replicates per condition) for the ATP and MMP assays, 2–6 replicates per condition (8–16 total replicates per condition) for the OCR assessment. Each experiment was normalized on the corresponding CellTracker Blue fluorescent signal. Values are shown as the percentage of the control condition. Student Unpaired t-test, *p < 0,05; ***p < 0,001. ATP: adenosine triphosphate, MMP: mitochondrial membrane potential, OCR: oxygen consumption rate

To validate our key data in cells expressing endogenous P301L‐tau, we used human iPSCs derived from a patient carrying the P301L‐tau mutation [20]. Cells were differentiated into neurons, and mitochondrial transplantation was performed with mitochondria isolated from iPSC-derived astrocytes. Key bioenergetic readouts were assessed 48 h after transplantation with 25 µg/mL mitochondria (the optimal time point in our SH-SY5Y model). We observed a 22% increase in ATP level (*p* < 0.001, Fig. 4a), an 18% increase in MMP (*p* < 0.001, Fig. 4b), and a 50% increase in OCR (*p* = 0.031, Fig. 4c) compared to the corresponding control condition.

These data demonstrate that mitochondrial transplantation also increased the mitochondrial bioenergetic function in patient (P301L mutant)-derived neurons.

### Mitochondrial transplantation enhances neurite outgrowth in both the Vector and P301L cells

We showed that mitochondrial transplantation improved the bioenergetic characteristics after 48 h of incubation with the recipient cells. Because mitochondrial bioenergetics is essential in building neuronal networks by providing the energy necessary for neurite formation, a morphological analysis of neurite outgrowth was performed on the Vector and P301L SH-SY5Y cells after two days of treatment with mitochondria. The characterization of the neurite outgrowth parameters of the P301L cells in comparison with the Vector is shown in Supplementary Fig. 3. In these experiments, nerve growth factor (NGF) was used as a positive control, and all neurite outgrowth parameters were measured after 48 h of treatment.

**Fig. 5:**
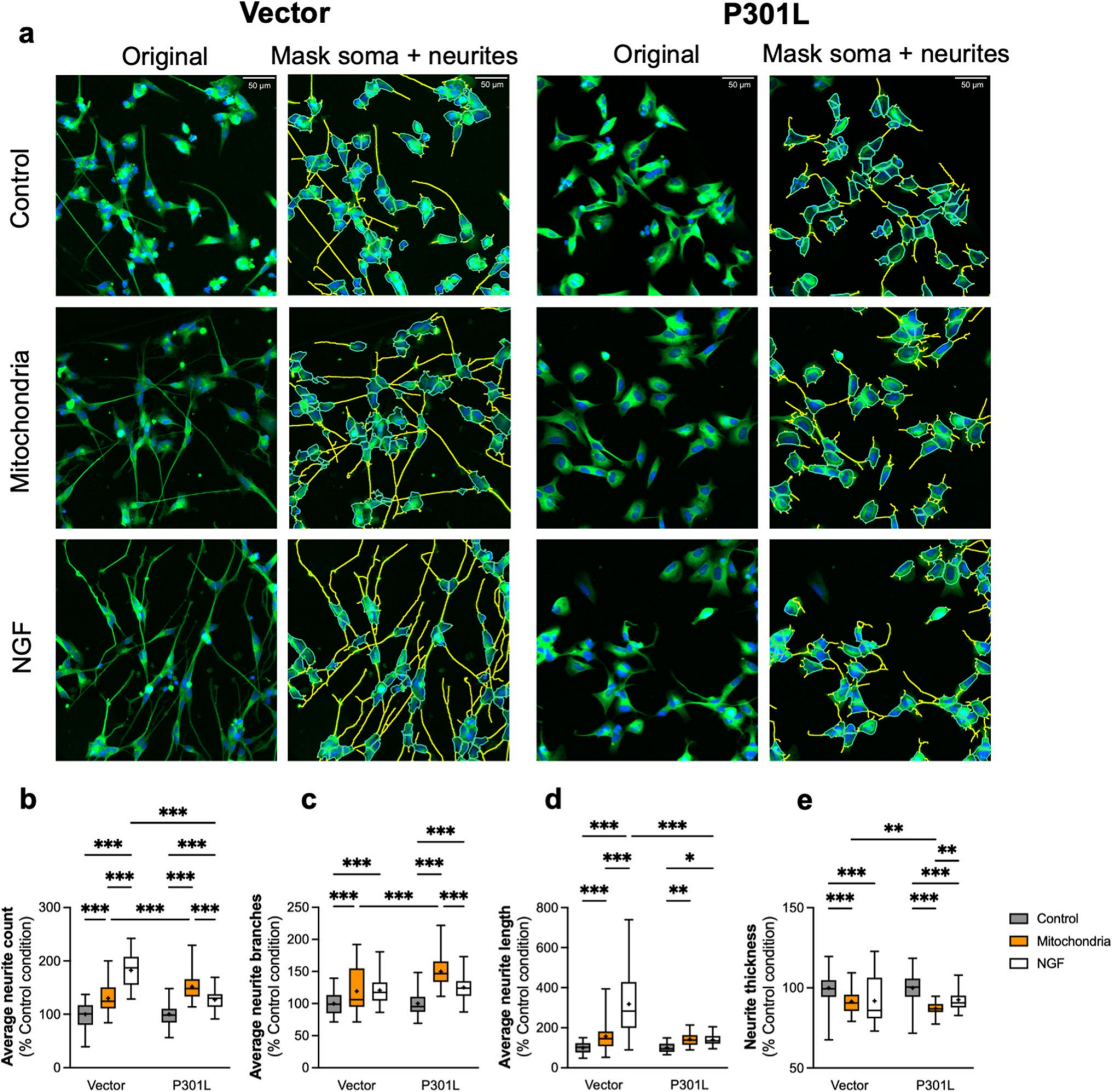
Impact of mitochondrial transplantation on neurites outgrowth parameters in the Vector and P301L SH-SY5Y cells. The « control» condition corresponds to the vehicle-treated condition with the mitochondrial assay solution. The « mitochondria» condition corresponds to treatment with 25 µg/mL of isolated mitochondria. The « NGF» condition corresponds to the positive control in which cells were treated with 50 ng/mL of NGF. (**a**) Cells were stained for ß3 tubulin (green color) and DAPI (blue color). All the pictures were captured 48 h post-transplantation at 20 × magnification. (**a**) Pictures of Vector and P301L cells were acquired with the Cytation 5, and the neurites analysis was performed with the neurites outgrowth module from Agilent. (**b**) Average neurite count per cell in Vector and P301L cells. (**c**) Average neurite branches in Vector and P301L cells. (**d**) Average neurite length in Vector and P301L cells. (**e**) Neurites thickness of Vector and P301L cells. The boxes represent the median (full line) and the mean (“ + ” symbol), the whiskers represent the minimal and maximal values. Each data set represents *N* = 3 independent experiments with 15–20 replicates per condition (40–50 total replicate number per condition). Values are shown as the percentage of the control condition. Two-way ANOVA and post hoc Tukey’s multiple comparison tests. *p < 0,05; **p < 0,01; ***p < 0,001. NGF: nerve growth factor

To measure several parameters characterizing neurite outgrowth, we used the Cytation 5 (Agilent) and the "Neurite Outgrowth Module", which allowed us to generate masks highlighting the soma and neurites of the neuronal cells (Fig. 5a). Neurite outgrowth parameters were automatically calculated by the Gen5 imaging software.

The Vector and P301L cells treated with the positive control nerve growth factor (NGF) presented an increase in the average neurite count (*p* < 0.001) (Fig. 5b), the average neurite branches (*p* < 0.001) (Fig. 5c), the average neurite length (*p* < 0.001 and *p* = 0.012) (Fig. 5d), and a decrease in neurite thickness (*p* < 0.001, Fig. 5e), indicating an enhanced neuronal differentiation and plasticity.

In the mitochondria-treated cell, we observed an increase in the average number of neurites and branches per cell in the Vector (+ 30% and + 19%, respectively) and P301L cells (+ 51% and + 50%, respectively) compared to the control condition (*p* < 0.001, Fig. 5b, c). In addition, neurite length was also increased after treatment in both cell line (+ 57% and + 42% for Vector and P301L cells, respectively) (*p* < 0.001 and *p* = 0.003) (Fig. 5d). Of note, the average neurite length observed after mitochondrial transplantation in P301L cells was as high as with the NGF treatment. In parallel, a decrease in neurite thickness was observed in the Vector and P301L cells compared to the control condition (−9% and −13%, respectively) (*p* < 0.001 for both) (Fig. 5e). Moreover, the mitochondrial transplantation showed better effects for the average neurite number, the average neurite branches and the neurite thickness on P301L cells compared to the Vector cells (+ 21%, + 31%, *p* < 0.001, and −5%, respectively, *p* = 0.003, Fig. 5b-c, e).

In conclusion, mitochondrial transplantation had a positive impact on the neuronal differentiation process for both Vector and P301L cells, which showed an increase in every neurite outgrowth parameter.

## Discussion

In this study, we hypothesized that mitochondrial transplantation could enhance the cellular viability, bioenergetic functions, and neurite outgrowth of both healthy and tau-mutant P301L cells.

In the first step, we optimized the mitochondria isolation protocol and determined the optimal treatment concentration. Our data indicate that the protocol used for mitochondrial isolation preserved mitochondrial function and integrity, as the mitochondria remained bioenergetically active after the process. Moreover, we demonstrated that isolated mitochondria can enter recipient cells.

A previous study by Shi et al. has shown that isolated mitochondria are found in recipient cells after only 30 min of incubation [14]. In line with our observations, we found that isolated mitochondria were present in the recipient cells after incubation for 24 h and 48 h. The putative mechanisms by which recipient cells internalize isolated mitochondria remain elusive. Studies have shown that endocytosis is the main entry pathway. However, the exact type of endocytosis (e.g., pinocytosis, clathrin- or actin-dependent endocytosis) is still debated [13]. In a study by Pacak and colleagues, the authors treated cardiomyocytes with inhibitors of clathrin-mediated endocytosis, actin-mediated endocytosis, or pinocytosis and assessed the entry of isolated mitochondria [27]. They found that isolated mitochondria enter cardiomyocytes through actin-dependent endocytosis. Similar investigations are currently being conducted in our group to determine which entry pathway isolated mitochondria use to enter SH-SY5Y cells.

Based on our microscopy experiments, we estimated that less than 30% of the SH-SY5Y cells were positive for transplanted mitochondria 24 h after transplantation. However, this number could be underestimated, depending on the fate of the transplanted mitochondria once they are in the recipient cells. If the isolated mito-RFP or mito-GFP mitochondria fuse with the endogenous network or are degraded in lysosomes, it can cause a decrease in their fluorescence intensity, subsequently leading to an underestimation of the number of cells that internalized them. It is also not excluded that some cells internalize several isolated mitochondria, while others do not internalize any.

An increasing body of literature has shown that intercellular mitochondrial transfer occurs naturally between cells and involves several distinct mechanisms, including tunneling nanotubes, extracellular vesicles, and the release of free mitochondria (reviewed in [28]). However, the mechanism of free mitochondrial uptake and the fate of exogenous mitochondria once inside recipient cells remain topics of ongoing debate. For instance, in a recent quantitative study, Al Amir Dache et al. demonstrated that isolated mitochondria enter HeLa cells via macropinocytosis-like fluid-phase uptake, with fewer than 10% escaping endosomes to reach the cytosol and potentially integrate into the host mitochondrial network [29]. Most internalized mitochondria were trafficked to lysosomes for degradation. Similarly, Lin et al. demonstrated that mitochondria from mesenchymal stromal cells (MSCs) were taken up by endothelial cells through clathrin-independent endocytosis, but did not integrate; instead, they triggered mitophagy via the PINK1–Parkin pathway, resulting in enhanced mitochondrial biogenesis [30]. In contrast, another study reported that exogenous mitochondria can escape endolysosomal compartments and successfully fuse with the endogenous network in human iPSC-derived cardiomyocytes, resulting in sustained increases in ATP production [31]. In line, Pacak et al. also found that transplanted mitochondria entered neonatal rat cardiomyocytes through actin-dependent endocytosis/phagocytosis, with evidence for long-term mtDNA transfer and respiratory rescue [27]. Conversely, a genome-wide CRISPR screen in BV2 microglia implicated heparan sulfate proteoglycans as necessary uptake factors, underscoring the diversity of entry pathways [32]. Together, these findings suggest that mitochondrial internalization and fate are highly variable, depending on the recipient cell type, donor source, and experimental context. This complexity underscores the need for further mechanistic studies to elucidate how transplanted mitochondria enter recipient cells—especially neurons, where the underlying processes remain unknown, particularly in disease-relevant conditions.

In the present study, after confirming that isolated mitochondria can indeed enter recipient cells, we demonstrated that mitochondrial transplantation was not toxic to the neuronal cells and had a positive impact on respiratory chain activity, as evidenced by increased ATP and MMP levels in undifferentiated cells. The BrdU assay revealed that the beneficial effects observed were not due to a proliferative effect of the treatment. Moreover, the slight decrease in cell proliferation observed after 48 h of treatment might be due to the modulatory effects of the mitochondrial transplantation on neuronal differentiation. Indeed, the promoting effect of mitochondrial transplantation on neuronal differentiation has been previously demonstrated in schizophrenia-derived induced pluripotent stem cells (iPSCs) differentiated into glutamatergic neurons [33]. These data are in line with the results obtained in our neurite outgrowth experiments. After this screening phase, we selected the 25 µg/mL mitochondria concentration for further experiments on differentiated SH-SY5Y cells.

Because undifferentiated SH-SY5Y cells are proliferative and not representative of mature neurons, the cells were differentiated to achieve a state as close as possible to that of neuronal cells [34]. Mitochondria transplantation was performed after differentiation of the Vector and the P301L SH-SY5Y cells. The cellular viability, ATP production, MMP levels, and cellular respiration were improved at 24 h and 48 h after the treatment for the Vector cells. The effects of mitochondrial transplantation on bioenergetic functions were more pronounced in differentiated cells than in undifferentiated ones. One explanation could be that the bioenergetic metabolism of undifferentiated and differentiated cells differs. Indeed, according to the Warburg effect, undifferentiated and proliferative cells exhibit a more glycolytic metabolism, whereas differentiated, non-proliferative cells primarily use the oxidative phosphorylation (OXPHOS) process [35]. Therefore, mitochondrial transplantation may enhance the respiratory activity of differentiated cells, as indicated by increased OCR, ATP production, and MMP. In addition, a decrease in the total and mitochondrial superoxide anion levels was observed after the mitochondrial transplantation, in line with the increased cellular viability. These positive effects of the transplantation are similar at 24 h and 48 h post-transplantation, suggesting that treatment might have durable effects. Ongoing experiments are currently being conducted in our group to investigate the long-term impact of mitochondrial transplantation in this cellular model and to determine the duration of the transplantation's effects. Specifically, we are conducting a longitudinal in vitro study that extends to 7 and 14 days post-transplantation.

The second main aim of this study was to determine whether mitochondrial transplantation could improve bioenergetic parameters in a cellular model of tauopathy that overexpresses the P301L-tau mutation in the MAPT gene [36]. This model is mainly associated with frontotemporal dementia but is also widely used as a general model of tauopathy. In particular, the P301L-tau mutation is related to abnormal hyperphosphorylation of the tau protein, as well as neuronal, synaptic, and mitochondrial dysfunctions. The P301L-tau overexpressing SH-SY5Y cells have been previously characterized in our laboratory, and their bioenergetic deficits compared to the Vector cells have been clearly demonstrated [16, 18]. We observed increased cellular viability, ATP levels, MMP, and OCR after treatment with mitochondria. The positive effects were overall maintained for at least 48 h. To strengthen the physiological relevance of our findings, we conducted additional experiments using iPSC-derived neurons carrying the P301L tau mutation. Mitochondria isolated from iPSC-derived astrocytes were transplanted into these human neuronal models. Our results indicate that mitochondrial transplantation significantly increases ATP levels, MMP, and OCR in P301L iPSC-derived neurons after 48 h, consistent with the effects observed in SH-SY5Y cells. These findings support the robustness of our bioenergetic data across two complementary in vitro systems. Ongoing experiments in our group aim to further characterize the effects of mitochondrial transplantation in this advanced human neuronal model.

Interestingly, while mitochondrial transplantation led to sustained increases in ATP production and OCR in P301L SH-SY5Y cells, the MMP returned to its baseline level after 48 h. This decoupling may suggest that energy output is supported by increased mitochondrial mass, rather than prolonged hyperpolarization. In parallel, the reduction in mitochondrial ROS observed 48 h post-transplantation points toward an adaptive response that preserves redox balance. A possible explanation is the feedback inhibition of the electron transport chain by high ATP levels or mild uncoupling mechanisms, which limits proton gradient buildup and prevents oxidative stress [37, 38]. In contrast, MMP remained elevated in Vector cells at 48 h, suggesting efficient integration of donor mitochondria in a non-pathological environment. It appeared that without mutant tau overexpression, Vector cells likely preserve mitochondrial dynamics and redox control, allowing stable membrane potential despite increased bioenergetic activity. Of note, P301L iPSC-derived neurons, which express endogenous levels of tau, displayed increased MMP at 48 h post-transplantation. This suggests that the decoupling observed in P301L SH-SY5Y cells may be linked to the overexpression of P301Ltau. This point definitely requires further investigation.

Different mechanisms may explain the improvement of bioenergetic parameters in our system.

either the effects are linked to the increase in mitochondrial mass in the recipient cells, or the increased mitochondrial efficiency per organelle. Based on the combined ATP, OCR, MMP, and ROS data, it can be hypothesized that mitochondrial transplantation enhances cellular bioenergetics through distinct mechanisms in Vector and P301L cells. Indeed, in Vector cells, the sustained elevation of ATP production, OCR, and MMP alongside reduced ROS after 48 h suggests improved mitochondrial efficiency per organelle, likely due to effective integration and functional coupling with endogenous mitochondria. In contrast, in P301L cells, where tau pathology disrupts mitochondrial dynamics and function [9, 17, 26], the persistence of elevated ATP and OCR despite normalized MMP at 48 h points toward an increased mitochondrial mass as the primary driver of rescue. The decline in mitochondrial ROS in both cell lines further supports the contribution of transplanted mitochondria in restoring redox balance.

Our results showing increased ATP production, enhanced oxygen consumption, and reduced ROS following mitochondrial transplantation are consistent with previously reported studies. For instance, Shi et al. (2017) demonstrated that mitochondrial transplantation on SH-SY5Y cells led to elevated ATP levels, improved complex I activity, and reduced apoptosis and oxidative stress after 24 h of treatment [14]. Similarly, Yang et al. [39] demonstrated that mitochondrial transplantation in Aβ-treated SH-SY5Y cells increased mitochondrial membrane potential (MMP), ATP levels, and antioxidant enzyme activity, while reducing reactive oxygen species (ROS) and Aβ aggregation [39]. These bioenergetic improvements were accompanied by upregulation of genes related to mitochondrial function and neuronal repair, suggesting a combination of enhanced mitochondrial function and improved cellular homeostasis. Such findings reinforce our hypothesis that mitochondrial transplantation contributes to bioenergetic improvement in P301L cells through both increased mitochondrial mass and enhanced efficiency of the mitochondrial network. Future experiments are needed to verify these claims, specifically by quantifying mitochondrial mass (e.g., via mtDNA quantification or citrate synthase activity), assessing oxidative stress markers such as 4-HNE, measuring antioxidant capacity, and evaluating mitophagy to help clarify the underlying mechanism.

We previously showed that P301L cells have a higher level of ROS than the Vector cells [26]. The transplanted mitochondria may have an antioxidant effect by bringing their own antioxidant system to the recipient cells, including manganese superoxide dismutase (Mn-SOD), which is located in the mitochondrial matrix [40]. It has also been shown that glutathione reductase levels increase after mitochondrial transplantation, suggesting that this enzyme may also enhance the antioxidant system in P301L cells [14].

Most of the bioenergetic properties of Vector and P301L cells have been positively affected by mitochondrial transplantation. Therefore, it was also interesting to focus on the morphological characteristics of the treated cells, such as neurite outgrowth. It has been demonstrated in a hybrid model of mouse neuroblastoma and rat glioma NG108-15 overexpressing the cDNA of human tau441, that tau cells have a higher number of neurites, and these neurites are thicker than in the wild-type cells [41]. In line with this, our P301L-tau overexpressing cells exhibit shorter neurites but more neurites per cell and a greater number of neurite branches than the Vector cells. Indeed, the hyperphosphorylated tau protein is known to destabilize the microtubules, which are involved in the elongation of neurites. The higher number of neurites and branches in the P301L cells may serve as a compensatory mechanism for the deficit in neurite length. It has been demonstrated in a previous study that mitochondrial transplantation can promote neurite growth in vitro in 6-OHDA-induced PC12 cells, serving as a model of Parkinson's disease [42]. Our results align with these findings, as we observe an increase in the average number of neurites and branches per cell for both the Vector and P301L cells. The neurite length was also enhanced in the P301L cells, suggesting that mitochondrial transplantation improves the neurite elongation capacity of these cells.

Taken together, our findings demonstrate that mitochondrial transplantation enhances bioenergetics functions and neuroplasticity processes in the context of tauopathies, making this therapeutic approach particularly relevant in tau-related neurodegenerative disorders.

The present study focuses primarily on phenotypic and functional outcomes, such as enhanced bioenergetics and neurite outgrowth, without delving into the mechanistic pathways that may underlie these effects. Importantly, the scope of this study was limited to mitochondrial function and neuronal morphology. We did not investigate other aspects of tau pathology such as aggregation, phosphorylation, or downstream signaling cascades. Indeed, as a first-step exploratory investigation, our objective was to evaluate whether mitochondrial transplantation could elicit a measurable functional increase in both healthy and energy-compromised (P301L) neuronal cells. Nevertheless, mechanistic insight is critical to advancing this approach toward in vivo application. While this was beyond the scope of our initial study, further analyses will aim to clarify whether mitochondrial transplantation modulates key mechanisms in tauopathy progression, including protein degradation, tau phosphorylation status, and autophagy/mitophagy.

In line with this idea, a recent study showed that mitochondrial transfer can activate mitophagy pathways, including PINK1/Parkin signalling and LC3-II induction, in endothelial cells [30]. Given that disease-associated tau impairs mitophagy by blocking Parkin translocation to depolarized mitochondria [9], it is plausible that mitochondrial transplantation may help restore mitophagy flux by bypassing this block or enhancing downstream clearance. Future studies assessing Parkin, LC3-II, and P62 levels post-transplantation will determine whether reactivation of mitophagy contributes to the observed functional rescue in P301L cells.

While our study demonstrates that mitochondrial transplantation leads to measurable improvements in bioenergetics and neurite outgrowth in vitro, there are obvious limitations to this simplified model. As discussed in our recent review [6], in vitro co-incubation may not accurately represent physiological uptake mechanisms and lacks key in vivo factors, such as immune surveillance, extracellular environment (e.g., extracellular calcium concentration), and vascular barriers (e.g., blood–brain barrier). These constraints underscore the importance of complementing in vitro findings with organotypic or in vivo models that better reflect the complexities of mitochondrial trafficking and immune interactions in the brain.

In vivo studies have shown that mitochondrial transplantation can enhance neuronal survival, reduce oxidative stress, and improve behavioral outcomes in models of Parkinson’s disease, stroke, and traumatic brain injury [6]

These effects have been achieved using various delivery methods, including direct intracerebral injection and intranasal administration, which overcome the limitations of in vitro co-incubation by enabling physiologically relevant uptake mechanisms.

The method of delivering mitochondria into the brain is also a crucial consideration. Indeed, a simple co-incubation, as done in our study, is not feasible in an in vivo context. Although the intracerebral injection looked efficient in some studies on animal models, it is also an invasive method. Other studies have used intravenous injection and found isolated mitochondria in various organs, including the brain, suggesting that isolated mitochondria can cross the blood–brain barrier [15, 43]. However, the method of mitochondria delivery should be more specific to target the brain precisely. The nasal route represents an interesting option because it is non-invasive and does not require a high amount of mitochondria [6, 44, 45].

The source of mitochondria also raises critical questions about immune compatibility. Interestingly, Ramirez-Barbieri et al. (2019), report that neither syngeneic nor allogeneic mitochondrial transplantation triggered direct or indirect alloreactivity in mice, and no evidence of damage-associated molecular pattern (DAMP) signaling was observed [46]. This held even after serial mitochondrial injections, suggesting that mitochondrial transplantation may be feasible without provoking innate or adaptive immune responses. These findings support the potential translational safety of mitochondria transplantation and reinforce the value of pursuing in vivo validation in tauopathy models, where immune context and delivery route will be central to therapeutic development.

Mitochondrial transplantation is increasingly explored as a therapeutic strategy in brain disorders, including ischemia, Parkinson’s disease, and traumatic brain injury. In our recent review [6], we summarized the accumulating evidence showing that mitochondrial transplantation can improve mitochondrial function, reduce oxidative stress, and support neuronal survival in both in vitro and in vivo models of brain disorders. Despite methodological differences across studies, such as delivery routes (intravenous, local, or intranasal), donor mitochondrial sources, and animal models, redundant beneficial effects are consistently reported, including the restoration of mitochondrial function, limitation of cellular damage, reduction of ROS, and enhancement of antioxidant responses. At the cellular level, mitochondrial transplantation was shown to modify gene expression, attenuate inflammation and apoptosis, and promote neurogenesis. These effects translate into improved cognitive and motor function, and reduced anxiety, depressive-like behaviors, and pain in preclinical models.

While promising, mitochondrial transplantation in brain diseases remains in early development compared to the cardiac field, where initial clinical studies have already shown safety and efficacy in pediatric patients with ischemic injury [47, 48]

Importantly, the first clinical trial of mitochondrial transplantation in the context of brain ischemia is currently underway (ClinicalTrials.gov: NCT04998357), representing a critical milestone in the clinical translation of this approach.

To our knowledge, no previous study has investigated the effects of mitochondrial transplantation in a tauopathy model. Here, we demonstrate that mitochondrial transplantation significantly enhances bioenergetic function and neurite outgrowth in both healthy SH-SY5Y cells and cells expressing the disease-associated P301L tau mutation. These findings support the concept of mitochondria-based interventions as a potential therapeutic strategy for neurodegenerative diseases involving mitochondrial dysfunction.

However, several limitations should be acknowledged. First, the primary model used in this study was the SH-SY5Y neuroblastoma line, which, while differentiated to adopt neuron-like properties, does not fully mimic the complexity of mature human neurons or the in vivo tauopathy environment. To address this, we have included preliminary key bioenergetic data using iPSC-derived neurons carrying the P301L mutation, thus enhancing the translational relevance of our observations. Further experiments are currently being conducted to investigate the effects of mitochondrial transplantation in greater depth using this advanced human neuronal model. Second, this study focused specifically on mitochondrial function and neuronal morphology (i.e., neurite outgrowth), and did not examine other hallmarks of tau pathology, such as abnormal phosphorylation, aggregation, or microtubule instability. Further work will be required to determine whether mitochondrial transplantation influences these processes. Lastly, validation of these findings in more physiologically relevant systems, including animal models of tauopathy, is needed to fully assess their clinical potential.

Taken together, our results suggest that mitochondrial transplantation represents a promising therapeutic strategy for enhancing neuronal bioenergetics and mitigating tau-related metabolic impairments. In this approach, mitochondria are not the target of the intervention—they are the therapeutic agent itself.

## Supplementary Information

Below is the link to the electronic supplementary material.

Supplementary file1 (MP4 183 KB)

Supplementary file2 (MP4 463 KB)

Supplementary file3 (MP4 2783 KB)

Supplementary file4 (MP4 4617 KB)

Supplementary file5 (DOCX 8959 KB)

## Data Availability

The corresponding data presented in this study are available on Open Science Framework (https://osf.io/2pju6/overview?view_only = 6f3983caf10546e881dbd2fa6cb70a98).

## Abbreviations

ADP: Adenosine diphosphate
ATP: Adenosine triphosphate
BrdU: Bromodeoxyuridine
BSA: Bovine serum albumin
DAMP: Damage-associated molecular pattern
DHE: Dihydroethidium
DMEM: Dulbecco’s modified Eagle medium
DMSO: Dimethyl sulfoxide
FBS: Fetal bovine serum
GFP: Green fluorescent protein
HRP: Horseradish peroxidase
HS: Horse serum
iPSCs: Induced pluripotent stem cells
MAPT: Microtubule associated protein Tau
MAS: Mitochondrial assay solution
MMP: Mitochondrial membrane potential
MT: Mitochondrial transplantation
MTT: 3-(4,5-Dimethylthiazol-2-yl)-2,5-diphenyltetrazolium bromide
NGF: Nerve growth factor
OCR: Oxygen consumption rate
PBS: Phosphate buffered saline
PFA: Paraformaldehyde
RFP: Red fluorescent protein
ROS: Reactive oxygen species
RPM: Revolutions per minute
RT: Room temperature
TMRM: Tetramethylrhodamine methyl ester
