# Supplementary material for: Mitochondrial Transplantation Increases Bioenergetics and Neurite Outgrowth in Healthy and P301Ltau-Expressing SH-SY5Y Cells
Source: Mol Neurobiol. 2025 Dec 10;63(1):279. doi: 10.1007/s12035-025-05604-y (PMC12695918; doi:10.1007/s12035-025-05604-y)
Supplement: Supplementary file 5 — Supplementary file5 (DOCX 8959 KB) [file 12035_2025_5604_MOESM5_ESM.docx]

**Supplementary Figures and Movies:**

**Supplementary Movies 1: Z-stack projection of isolated mitochondria in SH-SY5Y cells at 24h post-**

**transplantation.** SH-SY5Y cells were stained with the CellTracker blue dye (green false color), and the isolated

mitochondria from A172 cells expressing the mitoRFP tag are shown in red.

**Supplementary Movies 2: Z-stack projection of isolated mitochondria in SH-SY5Y cells at 48h post-**

**transplantation.** SH-SY5Y cells were stained with the CellTracker blue dye (green false color), and the isolated

mitochondria from A172 cells expressing the mitoRFP tag are shown in red.

**Supplementary Movies 3:** **3D Animation showing isolated mitochondria in SH-SY5Y cells at 24 hours post-transplantation.** SH-SY5Y cells were stained with the CellTracker blue dye (green false color), and the isolated mitochondria from A172 cells expressing the mitoRFP tag are shown in red. The 3D surface reconstruction and animation were obtained with the Imaris software.

**Supplementary Movies 4:** **3D Animation showing isolated mitochondria in SH-SY5Y cells at 48 hours post-transplantation.** SH-SY5Y cells were stained with the CellTracker blue dye (green false color), and the isolated mitochondria from A172 cells expressing the mitoRFP tag are shown in red. The 3D surface reconstruction and animation were obtained with the Imaris software.


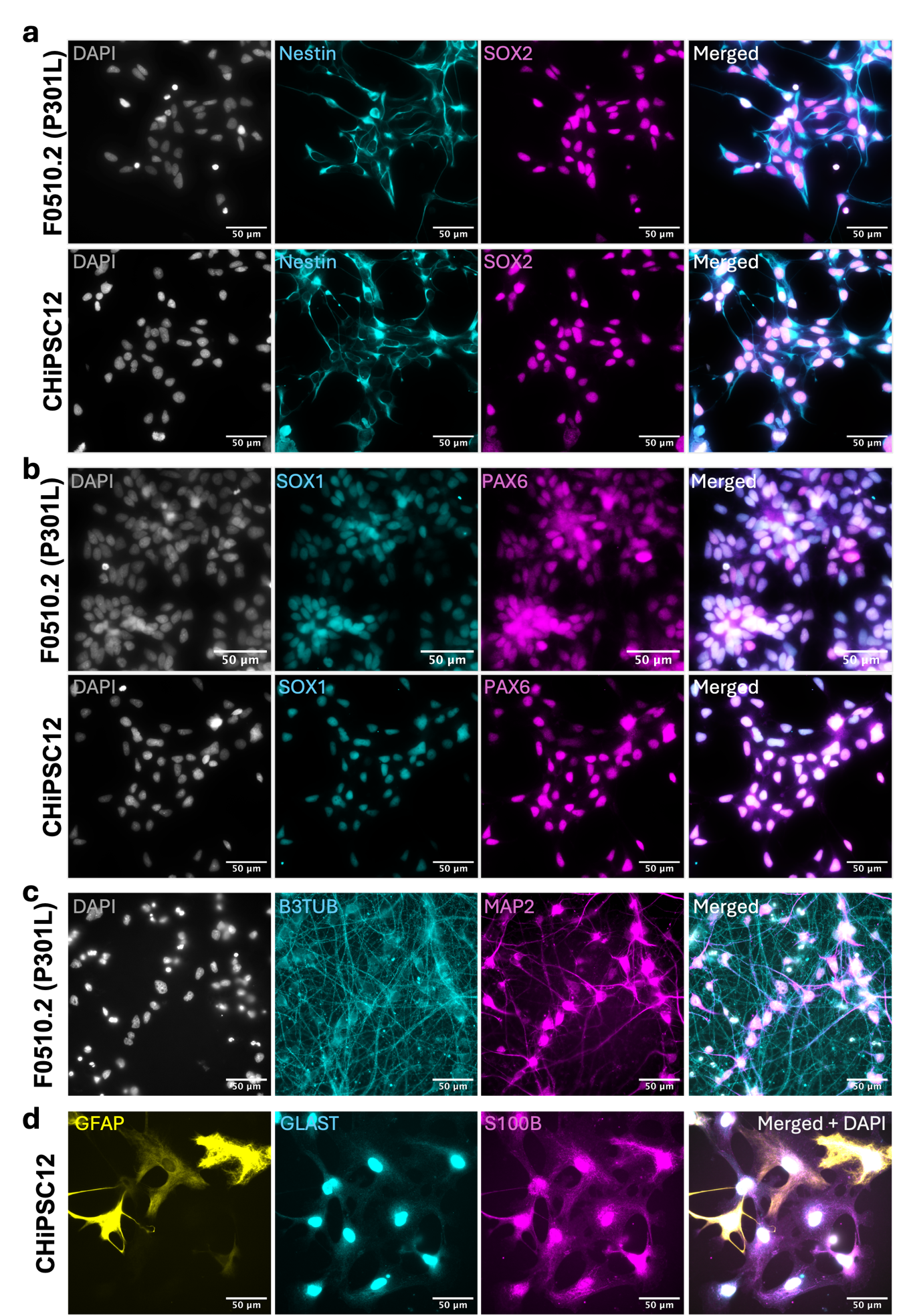


**Supplementary Figure 1: Quality control of IPSC-derived neuronal progenitor cells (NPC), neurons, and astrocytes.**

(a, b) Quality control of NPCs derived from F0510.2 (P301L mutant) and CHiPSC12 (control) iPSCs using the Neural Stem Cell Immunocytochemistry Kit, with staining for Nestin and SOX2 (a) and PAX6 and SOX1 (b). (c) iPSC-derived neurons express the neuronal markers B3TUB and MAP2. (d) iPSC-derived astrocytes express the astrocytic markers GFAP, EAAT1, and S100B. Scale bars represent 50 μm. B3TUB: ß3 tubulin, MAP2: Microtubule-Associated Protein 2, GFAP: glial fibrillary acidic protein, GLAST: glutamate aspartate transporter , S100B: S100 calcium-binding protein B

**
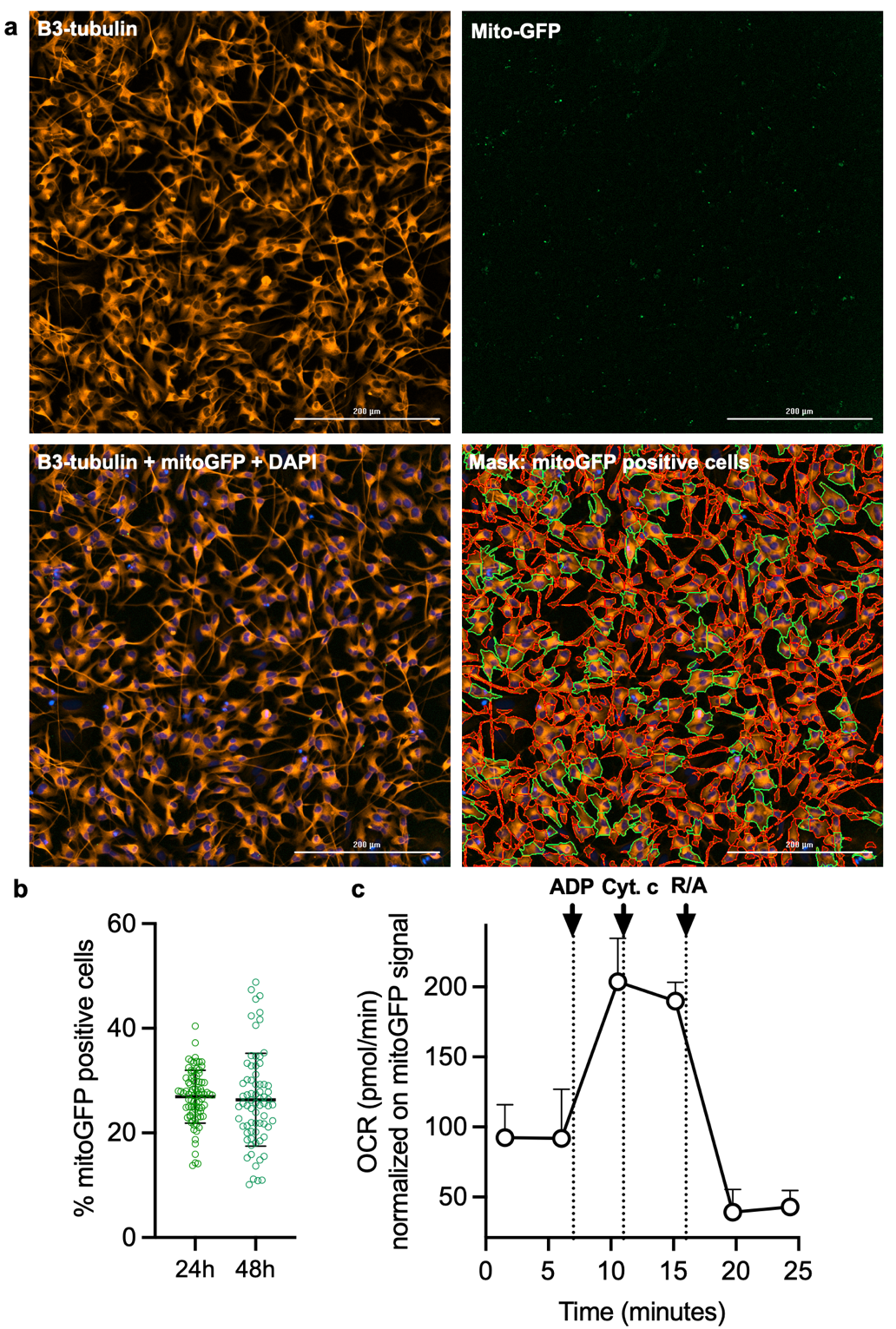
**

**Supplementary Figure 2: Isolated mitochondria entry into target cells and quality control.**

(a) Estimation of the proportion of SH-SY5Y cells that are positive for mitochondria from mitoGFP A172 cells. SH-SY5Y cells were co-incubated with GFP-tagged mitochondria (mitoGFP) from A172 cells. Cells were fixed and stained with β3-tubulin to visualize the cells’ area (in red) and DAPI to visualize nuclei (in blue). Images were obtained using the Cytation 5 (Agilent). The upper left picture shows SH-SY5Y cells stained with β3-tubulin (red color). The upper right picture shows the mitoGFP signal (green color). The lower left image is the merged image of the β3-tubulin staining, mitoGFP, and DAPI (nuclei in blue). The lower right image shows the masks generated by the Gen5 software (Agilent). The red mask highlights the SH-SY5Y cells without mitoGFP mitochondria; the green mask highlights the mitoGFP-positive SH-SY5Y cells. (b) Quantification of mitoGFP-positive SH-SY5Y cells 24 and 48 hours after transplantation. Data represent the percentage of SH-SY5Y cells that are positive for mitoGFP. Data are presented as the mean (black horizontal line), SEM, and individual values (one open circle = one microscopy image). In total, 72-84 images from 2 independent experiments were analyzed. (c) Oxygen consumption rate (OCR) was measured on freshly isolated mitoGFP mitochondria. After measurement of the basal respiration (state 2), adenosine diphosphate (ADP) was injected to trigger the respiratory state 3, followed by cytochrome c (Cyt. c) injection to assess the mitochondrial membrane integrity, and rotenone and antimycin A (R/A) to inhibit mitochondrial respiration. Data represent the OCR in pmol/min, normalized to the mitoGFP signal, and are presented as the mean and SEM of two independent experiments, with three replicates per experiment (total = 6 replicates).

| **** |
| --- |
| **Supplementary Figure 3: Characterization of the neurite outgrowth parameters on Vector and P301L SH-SY5Y cells.** (**a**) Average neurites count per cells of Vector versus P301L. (**b**) Average neurites branches of Vector versus P301L cells. (**c**) Average neurites length of Vector versus P301L cells (**d**) Neurites thickness of Vector versus P301L. The boxes represent the median (full line) and the mean (“ + ” symbol), and the whiskers represent the minimal and maximal values. Each dataset represents N=3 independent experiments *with 15-20 replicates per condition (40-50 total replicate number per condition)*. Values are shown as the percentage of the control condition. Student’s test; ***p<0,001. |
